# Fine sand facilitates egg extrusion and improves reproductive output in female mud crab genus *Scylla*

**DOI:** 10.7717/peerj.13961

**Published:** 2022-08-23

**Authors:** Hanafiah Fazhan, Khor Waiho, Alexander Chong Shu-Chien, Youji Wang, Mhd Ikhwanuddin, Muyassar H. Abualreesh, Nor Azman Kasan, Qingyang Wu, Sabri Muda, Chin Siang Sor, Mohamad Jalilah

**Affiliations:** 1 Higher Institution Centre of Excellence (HICoE), Institute of Tropical Aquaculture and Fisheries, Universiti Malaysia Terengganu, Kuala Nerus, Terengganu, Malaysia; 2 Shantou University-Universiti Malaysia Terengganu (STU-UMT) Joint Shellfish Research Laboratory, Shantou University, Shantou, Guangdong, China; 3 Centre for Chemical Biology, Universiti Sains Malaysia, Minden, Penang, Malaysia; 4 School of Biological Sciences, Universiti Sains Malaysia, Minden, Penang, Malaysia; 5 International Research Center for Marine Biosciences, Ministry of Science and Technology, Shanghai Ocean University, Shanghai, China; 6 Department of Marine Biology, Faculty of Marine Sciences, King Abdulaziz University, Jeddah, Saudi Arabia; 7 Key Laboratory of Applied Marine Biotechnology, Ministry of Education, Ningbo University, Ningbo, Zhejiang, China; 8 School of Marine Science, Ningbo University, Ningbo, Zhejiang, China; 9 Sayap Jaya Sdn Bhd, Selangor, Malaysia

**Keywords:** Broodstock culture, Sand size preference, *Scylla*, Crab farming, Egg extrusion

## Abstract

Mud crabs (genus *Scylla*) are gaining attention as potential aquaculture species due to their lucrative market price and high demand. One of the essential components of mud crab culture is broodstock quality. The optimisation of mud crab broodstock culture currently focused on its nutritional aspects and common rearing parameters, including stocking density and temperature. The use of sandy substrate to induce egg extrusion in female *Scylla* broodstock is common; however, its optimisation has never been conducted. This study investigated (1) the substrate association of two *Scylla* species (*S. olivacea*, *S. paramamosain*) during broodstock conditioning until egg extrusion when the choices of fine (66.99 ± 14.48 μm) and coarse (656.17 ± 38.13 μm) sands were given; (2) the female reproductive output when *S. olivacea* females were individually exposed to either fine sand, coarse sand, or no sand treatments. Females, spawners and non-spawners, of *S. olivacea* and *S. paramamosain* were associated with fine sand and none was observed to bury in the coarse sand tray. The occurrence of egg extrusion was not significantly different between species but moderately associated with the duration of visits (stayed in sand for 1 d, 2 d, ≥3 d). The final incubation period in the sand tray was more than 2 days in all cases, except for one female *S. paramamosain* (buried in the sand for 1 day prior to egg extrusion). When no choice was available, the highest percentage (58.3%) of females extruded eggs in fine sand treatment, followed by coarse sand treatment (33.3%), and no sand treatment (8.3%). Sand type influenced the weight of egg clutch, total egg number, fecundity, and clutch size. These results suggest that fine sand (<70 μm) substrate should be incorporated into *Scylla* broodstock rearing to maximise female reproductive output.

## Introduction

Mud crabs of the genus *Scylla* are intertidal crustacean species that contribute greatly to the fishery and aquaculture sectors of mud crab-producing countries, including China (Wu et al., 2020), Malaysia (Fazhan et al., 2022), Thailand (Moser et al., 2005), Philippines (Lebata et al., 2007), Indonesia (Fujaya et al., 2020), Myanmar (Segura-García, Yun & Box, 2018), Bangladesh (Bhuiyan et al., 2021), and countries in Southern Africa (Davis et al., 2004). The combined capture and aquaculture production of mud crab in 2019 was 51,139 t, of which all data were contributed by developing countries in Asia and Africa (FAO, 2022). Therefore, mud crab fishery plays crucial role in maintaining the livelihood of coastal communities, in addition to being one of the drivers of a country’s economic growth. There are currently four known species within the genus *Scylla* (*i.e*., *Scylla olivacea, S. paramamosain, S. tranquebarica, S. serrata*) (Keenan, Davie & Mann, 1998; Fazhan et al., 2020), of which most of them live sympatrically within the intertidal and subtidal zones of Indo-West Pacific region (Fazhan et al., 2017). The life cycle among *Scylla* species is similar, *e.g*., 10 embryonic development stages, five zoeal stages, one megalopa stage, and subsequent crablet stages (Ates et al., 2012; Waiho et al., 2018).

Despite their high market value, mud crabs are still not fully incorporated into aquaculture settings due to two main bottlenecks, the inconsistency in broodstock performance and unpredictability of larval quality and survival (Waiho et al., 2018). Most crab farms rely heavily on the supply of wild broodstock and crablets, and this puts tremendous stress on the already decreasing natural mud crab populations due to other potential anthropogenic factors (Ikhwanuddin et al., 2011; Waiho, Fazhan & Ikhwanuddin, 2016; Segura-García, Yun & Box, 2018; Viswanathan et al., 2019). The emerging demand for soft-shell crabs due to their lucrative price and short culture duration further escalates the matter, whereby increasing numbers of mud crab juveniles are being captured for soft-shell crab production (Hungria et al., 2017; Tavares et al., 2017; de la Cruz-Huervana, Quinitio & Corre, 2019; Waiho et al., 2021).

The development of mud crab seed production technology is tightly linked with broodstock selection and management (Azra & Ikhwanuddin, 2016). In crustaceans (Racotta, Palacios & Ibarra, 2003; Oniam, Chuchit & Arkronrat, 2012), including in mud crabs (Djunaidah et al., 2003), high-quality broodstock is directly reflected in the quality and quantity of larvae produced. Therefore, many efforts have been directed toward the understanding of the broodstock gonadal maturation process (Quinitio, Pedro & Parado-Estepa, 2007; Waiho et al., 2017; Wu et al., 2020) and the enhancement of broodstock quality, especially in the area of broodstock nutritional requirement and enhancement (Millamena & Bangcaya, 2001; Djunaidah et al., 2003; Ghazali et al., 2017; Hidir et al., 2018). However, an equally important aspect of broodstock culture, in addition to its diet and nutrition, is the optimisation of broodstock culture parameters. Thus far, besides the study by Saha et al. (2000) which focused on the effect of stocking density on broodstock, and Hamasaki (2003) which investigated the effect of temperature on egg and larvae development of *S. serrata*, limited studies have looked into the optimisation of *Scylla* broodstock culture parameters.

Several estuarine portunid species, such as those of genus *Callinectes* (Norse, 1977), *Portunus* (Yang et al., 2014), and *Scylla* (Hyland, Hill & Lee, 1984; Hill, 1994; Koolkalya et al., 2006) are known to migrate from coastal zones to deeper offshore waters during spawning season, and return to coastal zones after spawning. Such migration by euryhaline intertidal species such as mud crabs during spawning is thought to facilitate higher larval survival, a wider larval dispersal strategy and subsequently enable megalopae to recruit to new coastal habitats (Hill, 1994). The knowledge on female migration during spawning provides us with the preferred environmental factors such as temperature and salinity (Waiho et al., 2018). In addition, although different types of bottom substrates such as sand and gravel (Alava et al., 2007), pebbles and thick sand (Millamena & Quinitio, 2000), normal sand (Zeng, 2007; Baylon, 2009; Hidir et al., 2021), and fine coral sand (Hamasaki, 2003) have been used to facilitate egg extrusion in female mud crabs during broodstock culture in captivity, little is known about the substrate association during egg extrusion and the exact impact of sand type on female reproductive output.

Therefore, the aims of this study were to determine the substrate association of female mud crabs when presented with two types of sand substrates (fine sand, coarse sand) during egg extrusion, and to evaluate the impact of sand substrate on female reproductive output (clutch size, egg mass weight, egg number, fecundity, days to egg extrusion) when choice was not available. The results of this study would provide essential guidelines for the optimisation of an important culture parameter, *i.e*., the use of bottom substrate, during mud crab broodstock cultivation to maximise female reproductive output.

## Methodology

### General setup

Mature females of *S. olivacea* and *S. paramamosain* were obtained from local fishermen of Setiu Wetlands, Terengganu and transported live in dry conditions to the marine hatchery of the Institute of Tropical Aquaculture and Fisheries (AKUATROP), Universiti Malaysia Terengganu, Malaysia (approximately an hour distance by vehicle). Mud crabs are commercial species and thus no specific permit is required for their capture and purchase in Malaysia. Only healthy individuals with full limbs and no obvious abnormality or external parasites were used (Fazhan et al., 2018; Fujaya et al., 2020). Species identification was conducted according to Keenan, Davie & Mann (1998) and Fazhan et al. (2020). Female maturation status was determined based on their abdomen shape and coloration, *i.e*., mature female exhibits globular and darkened abdomen (Ikhwanuddin et al., 2011; Fazhan et al., 2021); ovarian maturation stage was assessed by probing *via* the transparent membrane between the posterior width of carapace (PWC) and the first abdomen segment, only healthy females with orange ovaries (ovarian stage II–III) were used (Quinitio et al., 2002; Wu et al., 2020). Once arrived in the hatchery, crabs were disinfected in 150 ppm formaldehyde for 30 min and subsequently subjected to a 3-day acclimatisation period (Baylon, 2011; Waiho et al., 2015). Their carapace width (CW) and body weight (BW) were measured and recorded. To ensure uniformity, only adult females with CW between 90–100 mm and BW above 175 g were used in this study (Waiho, Fazhan & Ikhwanuddin, 2016; Fazhan et al., 2017). All females were uni-eyestalk-ablated before subjected to both experiments. The general rearing protocols across treatments were as follow: temperature at 29–30 °C, 30–33 ppt, recirculating water system with weekly water exchange, and daily feeding at 0800 and 1,700 h with 5% body weight of mixed feed consisted of trash fish (*Selaroides* sp.) and squid (*Loligo* spp.) (Asmat-Ullah et al., 2021), unless stated otherwise.

### Experiment 1–substrate association in *S. olivacea* and *S. paramamosain*

Crabs were randomly stocked into a fiberglass tank of internal dimension 121.5 × 306 × 59 cm (width × length × height), with 16 females in one tank as one replicate, and duplicates were conducted for each species. Each crab was labelled using a waterproof silver marker on its carapace for easier identification. Eight trays of sand (four trays of fine sand and four trays of coarse sand) were placed evenly across each tank. The internal dimension of each tray was 36.5 × 50 × 9 cm (width × length × height), and sand was filled up to approximately 5 cm height. Fine sands were sourced from a beach next to the hatchery whereas coarse sands were purchased from an aquarium shop. All sands were rinsed with freshwater, immersed in formaldehyde for 1 h, and re-rinsed with freshwater 24 h before use. Right before being placed into tanks, each sand tray was rinsed with marine water to ensure that the salinity within sand particles was similar to that used in the culture system. Sand particles were randomly collected and at least 150 sand particles of each sand type were measured under Nikon Eclipse 80i microscope. Crabs were reared in the system for 30 days. Sand trays were removed from the culture system and replaced with clean disinfected sand trays every 3 days. Used sand trays were cleaned by removing any faeces or food particles prior to cleaning with freshwater and disinfecting with formaldehyde as mentioned above. Daily observation was made an hour after morning feeding at 0900 h. Crabs that were partially (50%) or fully buried in the sand were recorded. Females were immediately removed from the culture system once they have extruded eggs onto their abdomens. ‘Days of visit’ indicates the continuous period of staying buried in the sand. For example, a crab can exhibit multiple ‘1 d’ visits and ‘2 d’ visits prior to egg extrusion.

### Experiment 2–egg extrusion under unfavourable conditions

Based on the results of Experiment 1 whereby all females extruded eggs only on fine sand, we further characterised the effect of sand on female egg production. Only females of *S. olivacea* were used in this experiment. In this experiment, females were individually subjected to three treatments, (i) fine sand, (ii) coarse sand, and (iii) no substrate. Each treatment was made up of 12 individuals and each individual was cultured in a 45 × 30 × 45 cm (width × depth × height) transparent aquarium and covered with a black covering. A tray of similar dimensions (width and depth) was used to place either fine sand or coarse sand into each aquarium. Due to the lack of recirculating water system in this setup, 50% water exchange was conducted every 2 days and full water exchange was conducted on day 8. Other rearing parameters were as described in 2.1. Similar to in 2.2, sand trays were removed and disinfected every 3 days. Crabs were cultured for a period of 30 days or until eggs were extruded. Females were removed from the culture system right after egg extrusion. The clutch fullness of each female was determined *via* visual determination, with 100% clutch fullness indicating an abdomen that is completely filled with egg mass (Jadamec, Donaldson & Cullenberg, 1999; Danielsen et al., 2019). Clutch fullness index (CFI) was estimated by visually assigning gravid females into six categories based on a modified description by Militelli et al. (2019): 100% full, 75% full, 50% full, 25% full, traces to 12.5% full, and empty. The weight of egg mass was estimated by subtracting the body weight of females before and after egg extrusion. Fecundity was estimated by dividing the weight of egg mass by the average individual egg mass approximated from the number of eggs in 0.01 g; three subsamples of 0.01 g from a random area of the egg mass were collected as replicates (modified from Krimsky et al. (2009)).

### Data analysis

All data were analysed using IBM SPSS Statistic ver.25 (SPSS, Inc, Chicago, IL, USA). Homogeneity of variance and normality of data were checked using Levene’s test and Shapiro–Wilk test, respectively. The relationship between (1) number of days staying in the sand tray and egg extrusion occurrence, (2) species and frequency of visit, and (3) the prevalence of egg extrusion occurrence with sand types were investigated using Chi Square test for association. Due to the low frequency of egg extrusion occurrence in the category of ‘stayed in sand for ≥3 d’ of *S. olivacea*, only two categories (*i.e*., stayed in sand for 1 d, ≥2 d) were used in subsequent Chi Square tests to obtain the expected cell frequencies of greater than five. The strength of association between significant variables was tested using Phi (φ) (for 2 × 2 crosstabulation) and Cramer’s V (for other than 2 × 2 crosstabulation) (Conover, 1999). Fisher’s Exact test was conducted between sand type and clutch size due to the small sample size (expected cell frequencies were less than five (Blalock, 1999)). Poisson regression analysis was performed to test the interaction between the number of spawners and two independent variables (the final incubation days and species). The data of days to extrusion of both species were normal, as assessed by Shapiro–Wilk test (*p* > 0.05) and there was homogeneity of variances for days to extrusion for *S. olivacea* and *S. paramamosain*, as assessed by Levene’s test for equality of variances (*p* = 0.639). Independent Sample T-test was used to compare (1) sand particle size, and (2) the number of days to extrusion of females between the two species. Pearson’s Chi-Square goodness-of-fit test was used to determine whether egg extrusion will be more prevalent in fine sand compared to coarse sand or no sand at all. Data of weight of egg clutch, fecundity, the total number of eggs, and days-to-extrusion were normal (*p* > 0.05) but One-Way Analysis of Variance (ANOVA) with Welch correction was conducted when investigating the effect of sand type on each dependent variable to account for the unequal sample size. The ‘no sand’ treatment was omitted from the comparison owing to its low sample number (*i.e*., only one female extruded egg in the treatment). All reported data are expressed in mean ± standard deviation unless otherwise stated.

## Results

### Spawning characteristics

All spawned females extruded eggs either partially buried in the fine sand tray, sitting on top of the given substrate, or swimming in the water column above the given substrate. During the egg extrusion process, the female was observed to extend her walking legs, abdomen flap opened outwards, swimming legs pointing out, and the pleopods were moving in a wave-like motion as eggs were extruded. Some eggs were seen to be scattered around the female, and she would collect some of the scattered eggs from the sand substrate by slightly lowering her abdomen onto the bottom surface, enabling her pleopods to be in contact with the eggs.

### Substrate association

**Figure 1:**
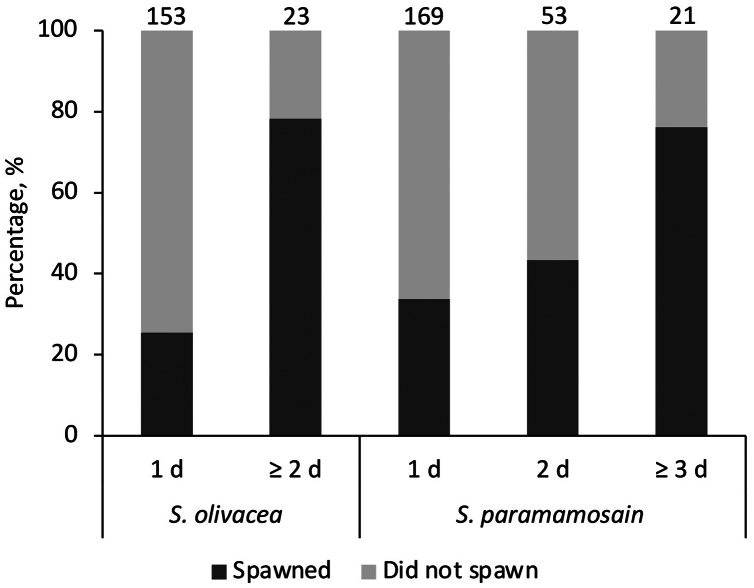
The percentage of females that have successfully spawned and did not spawn, categorised by the stayed duration in the sand tray (1 d, 2 d, or ≥3 d) and species *Scylla olivacea* and *Scylla paramamosain*. Note that for *S. olivacea*, the category ‘≥3 d’ was collapsed and merged with ‘2 d’ to ensure the assumptions of the Chi Square test were observed. The number above each bar represents the total number of females in each category.

The sand particle size of coarse sand used in this study was approximately 10 times larger than those of fine sand (*t*(191.10) = 105.12, *p* < 0.001; average fine sand particle size = 66.99 ± 14.48 μm, average coarse sand particle size = 656.17 ± 38.13 μm). When given preference as in Experiment 1, all females (spawners or non-spawners) preferred fine sand regardless of species. No female was observed to bury in the coarse sand tray. The average egg extrusion percentages of *S. olivacea* and *S. paramamosain* in Experiment 1 were 37.5 ± 0.09% and 46.9 ± 0.04%, respectively. All expected cell frequencies were greater than five when comparing between species and days of visits, meeting the required assumption for Chi Square test. There was no statistically significant association between species (*S. olivacea*, *S. paramamosain*) for egg extrusion occurrence, χ^2^(2) = 1.624, *p* = 0.444 (Fig. 1). Egg extrusion occurrence was significantly associated with the days of visits in *S. olivacea* (χ^2^(1) = 25.427, *p* < 0.001) and *S. paramamosain* (χ^2^(2) = 14.522, *p* = 0.001) (Fig. 1). There was a moderately strong association between duration of visits and egg extrusion occurrence in both species, with that of *S. olivacea* exhibiting a stronger association (φ = 0.380, *p* < 0.001) than *S. paramamosain* (φ = 0.244, *p* = 0.001). Furthermore, in all cases of both species that have successfully spawned, the final incubation period in sand was more than 2 days, and only one *S. paramamosain* female spawned after burial in the sand tray for a day. However, based on the Poisson regression, the number of spawners was not significantly affected by the final incubation days (χ^2^(4) = 5.674, *p* = 0.225) or species (χ^2^(1) = 0.610, *p* = 0.435). The number of days to extrusion was similar between *S. olivacea* females (average days to extrusion: 20.42 ± 6.64 days) and *S. paramamosain* females (average days to extrusion: 20.93 ± 6.88 days), *t*(25) = 0.197, *p* = 0.846.

### Egg extrusion under unfavourable conditions

**Figure 2:**
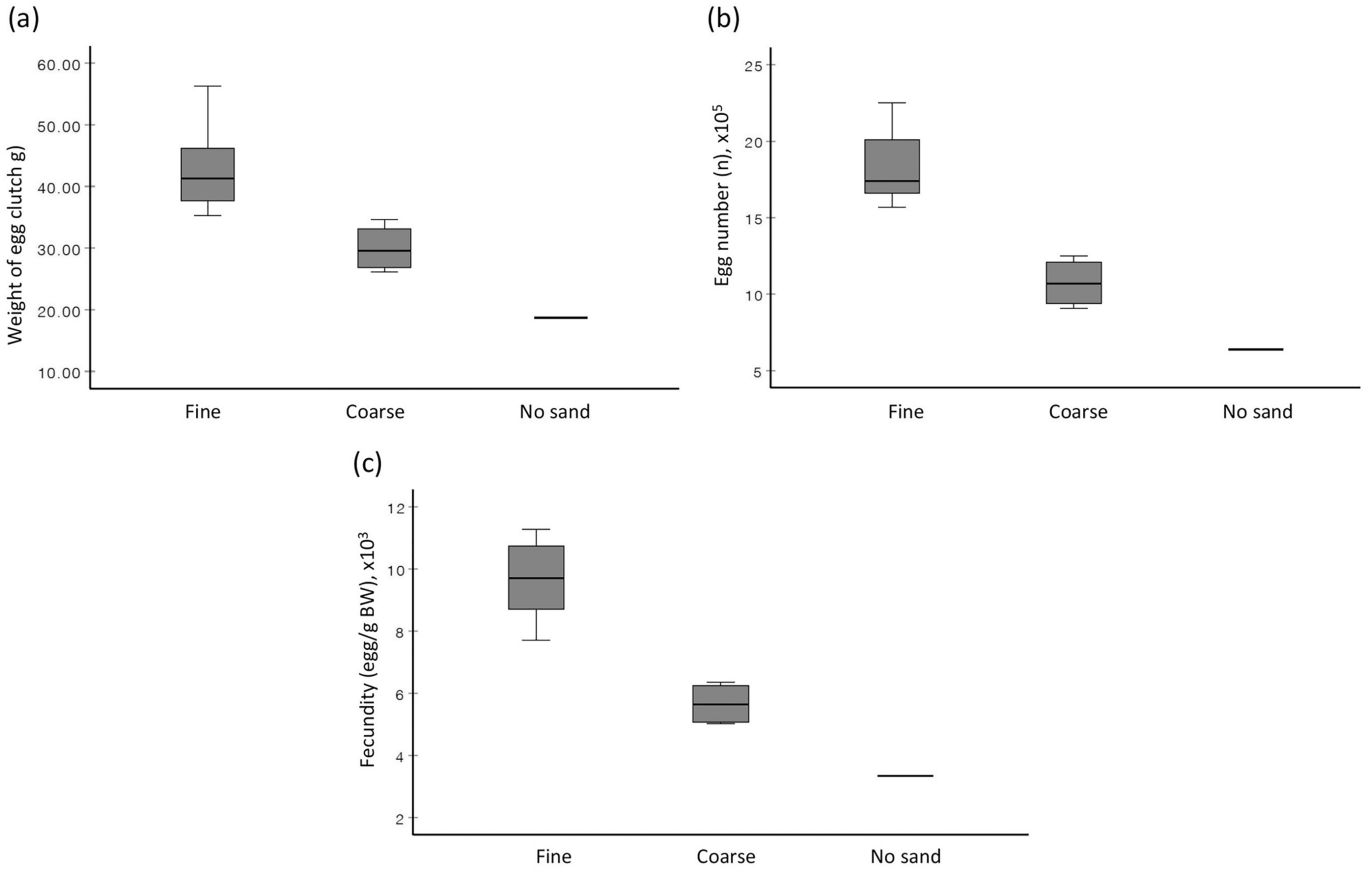
Boxplots depicting the (A) average weight of extruded egg mass, (B) average total egg number, and (C) average fecundity of *S. olivacea* females subjected to three different treatments. Treatments are ‘fine sand’ treatment, ‘coarse sand’ treatment, and ‘no sand’. Note: Only one sample in ‘no sand’ treatment.

When no choice was given (Experiment 2), the prevalence of egg extrusion among females of *S. olivacea* was significantly associated with sand types (χ^2^(2) = 6.750, *p* = 0.034), with the highest egg extrusion percentage observed in fine sand treatment (58.3%), followed by coarse sand treatment (33.3%), and no sand treatment (8.3%). The provided sand type (fine sand *vs* coarse sand) significantly affected the weight of egg clutch (F(1,9) = 14.812, *p* = 0.004), total egg number (F(1,8.8) = 37.962, *p* < 0.001), and fecundity (F(1,9) = 42.162, *p* < 0.001) (Fig. 2). Females, when reared in fine sand, extruded eggs significantly faster (F(1,9) = 2.503, *p* = 0.006) than those reared in coarse sand (Table 1). The association between sand type (fine sand *vs* coarse sand) and female clutch size (based on CFI) was statistically significant based on Fisher’s exact test (*p* = 0.04) (Table 1). In short, female mud crabs extruded higher egg quantity, exhibited better clutch size, and in shorter rearing duration when cultured in fine sand compared to in coarse sand.

**Table 1:** The clutch size range, average days to extrusion, and the number of spawned females of *S. olivacea* in different sand treatments.

	Treatment		
	Fine sand	Coarse sand	No sand*
Clutch size range	75–100% full	traces to 50% full	traces to 12.5% full
Average days to extrusion	16.86 ± 4.38	24.25 ± 2.50	28
Number of spawned females	7	4	1

**Note:**

* Only one sample in ‘no sand’ treatment.

## Discussion

The use of sandy substrates in broodstock development of most commercially important brachyuran species is common, as it is thought that it would provide refuge, aid in egg extrusion and clutch development, and minimise egg loss throughout the incubation period (Davis et al., 2004; Ravi & Manisseri, 2013). For example, Hamasaki & Fukunaga (2008) observed that depression in the sand facilitates the attachment of eggs to the abdominal pleopods of females of the swimming crab *Portunus trituberculatus*. However, little is known about the sand substrate association and impact of different sand types on female reproductive output. Females of both *Scylla* species (*S. olivacea* and *S. paramamosain*) selectively preferred to seek refuge and stay buried in fine sand compared to coarse sand. The avoidance of coarse sand might be due to the higher energy requirement needed by crabs to dig into compared to the fine sand substrate. Aquaculturists are known to make use of this knowledge and use sandy bottoms as an effective way to alleviate cannibalism in crab culture (Syafaat et al., 2021; Zhang et al., 2021).

The process of egg extrusion in *Scylla* females is strongly associated with longer days of visits, whereby females close to egg extrusion would stay buried for a longer period (≥2 d). It is postulated that the longer period prior to egg extrusion might be linked to the internal physiological changes in preparation for the egg extrusion process. Although the involvement of various hormonal peptides during ovarian maturation has been studied (Gong et al., 2016; Zeng et al., 2016; Kornthong et al., 2019), the potential role of regulatory proteins right before the egg extrusion process is worthy of investigation as it would be useful for the future development of spawning synchronisation technique in mud crab aquaculture. The average days to extrusion of *S. olivacea* and *S. paramamosain* found in this study provides a baseline estimation of culture days for mud crab broodstock culture in captivity, from ovarian stage II and above to egg extrusion.

The effects of different sand types on female reproductive output were highlighted when females were exposed to only one substrate throughout the culture period. The presence of sand substrate is utterly essential for egg extrusion in mud crabs, and fine sand provided better broodstock reproductive output compared to coarse sand. This is because when fine sand was the bottom substrate, the fallen eggs during the egg extrusion process remained near the female and aggregated on the surface of the sand substrate, thereby enabled their easy recollection onto the pleopods by the female broodstock after the whole extrusion process. Comparatively, coarse sand substrate complicated the egg re-collection process due to their large particle size. The scattering of eggs during egg extrusion was the most prevalent when no bottom substrate was available. This scenario was employed by Zeng (2007) to obtain high number of eggs without the need to isolate eggs from egg masses attached to female’s pleopod.

The substrate association of mud crab females for fine sand substrates to aid the egg extrusion process is also highly linked with their offshore migratory behaviour for spawning purposes. The offshore migration of *Scylla* spp. can go up to 50 km (Moser et al., 2005; Koolkalya et al., 2006), and this migration route often involves a change in bottom substrate, with more availability of sandy substrate. Therefore, in addition to increasing larval survival percentage and dispersal range (Hill, 1994; Ogawa et al., 2012), the migration of *Scylla* females may also be co-driven by the need for sandy substrate during the egg extrusion process. Future tag-recapture research of *Scylla* spp. and the detailed environmental characterisation of the location where berried females (females carrying egg masses) are found will provide sufficient support to this postulate.

Clutch size directly correlates to larval production quantity and fecundity in crabs (with low CFI scores had low fecundities and *vice versa*), and CFI has been proposed as a tool to understand the spatial and temporal changes in egg production of southern king crab *Lithodes santolla* (Militelli et al., 2019). Although comparatively less detailed as manual calculation of egg numbers and fecundity under a microscope, the assignment of female clutch size using a visual scale is also feasible in mud crabs, as evident in this study. The average number of eggs per clutch when mud crab females were subjected to fine sand treatment in this study was within the expected range of number of eggs reported in other studies (Koolkalya, Matchakuea & Jutagate, 2016; Viswanathan et al., 2019). The relative fecundity per batch of *S. olivacea* found in this study was comparable to that of *S. serrata* (10,655 ± 4,069 eggs/g) (Davis et al., 2004) and *S. paramamosain* (7,687 ± 1,812 eggs/g) (Djunaidah et al., 2003). As fecundity is positively correlated with portunid’s body size (CW) (Graham et al., 2012), only females of a small CW range were used in this study to better highlight the potential influence of sand type onto female reproductive output. The significantly reduced female reproductive output (*i.e*., smaller clutch size, egg weight, total egg number and fecundity) in females subjected to coarse sand treatment reflected the importance of sand choice during mud crab broodstock conditioning and culture. More importantly, sand should always be available during mud crab broodstock culture as the lack of it would not be favourable for *Scylla* females to extrude eggs.

Additionally, females extruded eggs in shorter time when subjected to fine sand treatment compared to those in coarse sand treatment. The significantly reduced time until egg extrusion during culture might be due to the conduciveness of the environment, that is, the presence of fine sands. Other environmental factors such as temperature, salinity, and nutrition can be safely excluded as they were being controlled throughout treatments in this study. Adverse conditions are known to delay ovarian development (Zheng et al., 2020; Luo et al., 2021) and even cause ovary resorption (Becker et al., 2020) in crustaceans. As sand is also an important substrate to reduce stress and minimise the build-up of fouling organisms in mud crabs (Becker & Wahl, 1996; Lavilla-Pitogo & De la Peña, 2004), the opportunity to be able to bury into the fine sand treatment might alleviate stress in females, thereby enhancing the egg extrusion process. In addition, some bacteria serve functional roles in egg defense of crustaceans during the extended egg-carrying period (Cawthorn, 2011), such as against various fungal infections that may cause fouling and disease (Nyholm, 2020). Future research on the change in bacteria composition of females’ abdomen cavity and on egg clutches, with or without sand treatment, would further support the beneficial effect of sand on mud crab’s general wellbeing and specifically, female broodstock’s reproductive output. Based on the significantly shorter culture time needed until the egg extrusion process in *Scylla* females when fine sand instead of coarse sand (or no sand) was used, it is therefore highly recommended that farmers and aquaculturists incorporate the usage of fine sand of less than 70 μm in mud crab broodstock culture.

## Supplemental Information

10.7717/peerj.13961/supp-1 Supplemental Information 1 Raw data for sand size, Experiment 1, and Experiment 2.

## Additional Information and Declarations

### Data Availability

The following information was supplied regarding data availability: The raw measurements are available as a Supplemental File.
